# Ultrathin tunable terahertz absorber based on MEMS-driven metamaterial

**DOI:** 10.1038/micronano.2017.33

**Published:** 2017-08-28

**Authors:** Mingkai Liu, Mohamad Susli, Dilusha Silva, Gino Putrino, Hemendra Kala, Shuting Fan, Michael Cole, Lorenzo Faraone, Vincent P. Wallace, Willie J. Padilla, David A. Powell, Ilya V. Shadrivov, Mariusz Martyniuk

**Affiliations:** 1 Nonlinear Physics Centre, Research School of Physics and Engineering, Australian National University, Canberra, ACT 2601, Australia; 2 School of Electrical, Electronic and Computer Engineering, the University of Western Australia, Crawley, WA 6009, Australia; 3 School of Physics, University of Western Australia, Crawley, WA 6009, Australia; 4 Department of Electrical and Computer Engineering, Duke University, Durham, NC 27708, USA

**Keywords:** absorber, metamaterials, micro-electro-mechanical system, terahertz, tunable device

## Abstract

The realization of high-performance tunable absorbers for terahertz frequencies is crucial for advancing applications such as single-pixel imaging and spectroscopy. Based on the strong position sensitivity of metamaterials’ electromagnetic response, we combine meta-atoms that support strongly localized modes with suspended flat membranes that can be driven electrostatically. This design maximizes the tunability range for small mechanical displacements of the membranes. We employ a micro-electro-mechanical system technology and successfully fabricate the devices. Our prototype devices are among the best-performing tunable THz absorbers demonstrated to date, with an ultrathin device thickness (~1/50 of the working wavelength), absorption varying between 60% and 80% in the initial state when the membranes remain suspended, and fast switching speed (~27 μs). The absorption is tuned by an applied voltage, with the most marked results achieved when the structure reaches the snap-down state. In this case, the resonance shifts by >200% of the linewidth (14% of the initial resonance frequency), and the absolute absorption modulation measured at the initial resonance can reach 65%. The demonstrated approach can be further optimized and extended to benefit numerous applications in THz technology.

## Introduction

Metamaterials and metasurfaces open unprecedented possibilities in manipulating electromagnetic waves at the subwavelength scale^1,2,3^. This possibility is enabled by the strongly localized resonances of the building blocks, ‘meta-atoms’, which offer new degrees of freedom in controlling not only the electric but also the magnetic component of waves, achieving electromagnetic properties unavailable with naturally occurring materials. Due to the limited intrinsic response of natural materials, metamaterials operating in the terahertz (THz) frequency band are of particular interest^4,5,6,7,8,9,10,11^.

Metamaterials can exhibit very strong absorption in layers impedance matched to free space^8,12,13,14,15^. The realization of metamaterial absorbers in THz frequencies allows more efficient energy harvesting for imaging and sensing to be performed in planar structures, which are suitable for integrated devices. For applications such as single pixel imaging or spectroscopy, two key figures of merit are the modulation index and the modulation speed.

The modulation of absorption can be accomplished by tuning the resonance frequency within a band of interest. Although the mechanism of resonant perfect absorption can be understood in a number of ways^12,14,16^, there is no general recipe for constructing ideal THz tunable absorbers. For a high-performance tunable THz metamaterial absorber, the following criteria should be satisfied: (1) it provides near-perfect absorption within the desired frequency band; (2) the resonant wavelength is strongly tunable; (3) the modulation speed is fast, particularly for imaging and spectroscopy applications for which this parameter determines the acquisition time; and (4) the absorber is electromagnetically thin, thus enabling device integration for advanced applications.

Among the numerous solutions for reconfigurable THz metamaterials and meta-devices (see Refs. 17,18 and references therein), the hybridization of microelectromechanical systems (MEMS) and THz metamaterials is one of the most promising paradigms. Compared to other tuning mechanisms that require electrical current, thermal or optical stimuli, tuning electrostatically with MEMS can substantially reduce thermal noise and energy consumption, which is crucial for sensing and imaging weak THz signals.

Although a number of THz tunable transmission filters based on MEMS-driven metamaterials have been demonstrated^19,20,21,22,23,24,25,26,27,28,29,30,31^, the realization of a high-performance MEMS-driven tunable THz absorber is still challenging, and only a limited number of demonstrations have been reported so far^32^. This low number is primarily due to the severe mismatch between the actuation range of most MEMS (on the scale of 1–10 μm) and THz wavelengths on the scales of 100–1000 μm. For absorbers based on Fabry–Pérot resonances^33,34,35^, the shift of the resonant wavelength *δλ*_0_ is on the same scale as the mechanical displacement of mirrors *δd*; therefore, it is very difficult to achieve a large resonance shift by deforming an electromagnetically thick THz absorber. However, it has been demonstrated that the near-field coupling of meta-atoms can have a substantial influence on the resonant frequency^36,37,38,39^; therefore, metamaterial THz absorbers based on matching localized electric and magnetic resonances are ideal to maximize the mechanical sensitivity of the resonance (*δλ*_0_/*δd*»1)^8,12,13,14, 15,40^, particularly when the thickness of absorber is reduced to the deep subwavelength scale to match the actuation range of MEMS.

Here, we design and experimentally demonstrate ultrathin (∼*λ*_0_*/*50) tunable THz absorbers. We combine meta-atoms that support strongly localized modes with flat, suspended silicon nitride membranes that can be driven electrostatically. Unlike tunable absorbers based on tilted cantilevers^32,41^, flat membranes can maximize the coupling between meta-atoms and the ground plane and enhance the modulation contrast by avoiding resonance broadening due to tilting. Our prototype devices show very good performance: strong absorption (60–80% at the initial state when the membranes remain suspended), fast switching speed (switching time ∼27 μs), highly enhanced mechanical sensitivity (*δλ*_0_*/δd* can be up to 14.8 when the device is driven to snap-down), and large modulation contrast (up to 65% of absolute change in absorption at the initial resonance under the snap-down state). Our study provides a practical pathway toward high-performance tunable absorbers, and the approach can be further extended to benefit numerous applications in THz and even infrared technology^42,43^.

## Materials and methods

**Figure 1:**
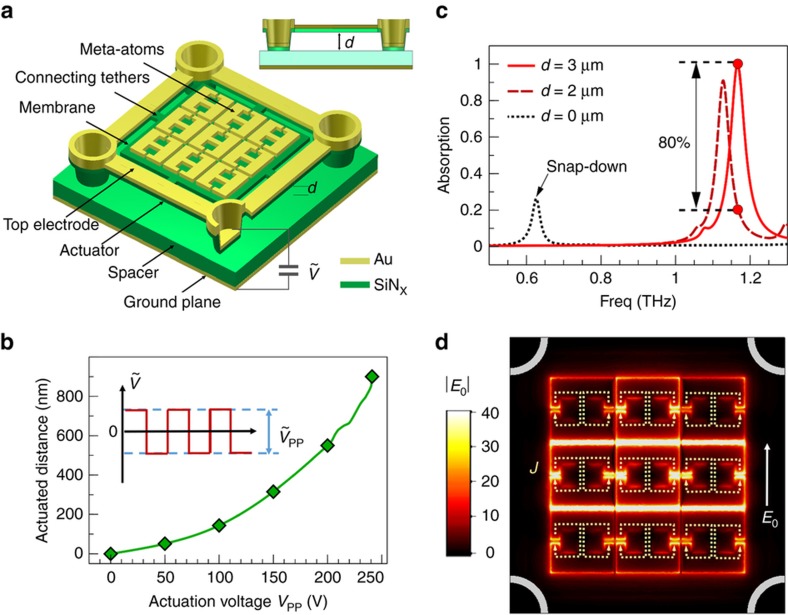
(**a**) Schematic of the unit cell of the THz absorber. The vertical distance between the meta-atoms and the ground plane can be tuned electrostatically. The shown element thicknesses are not to scale. (**b**) Measured MEMS actuation distance as a function of actuation voltage of an example device actuated with a square wave AC signal (*V*_pp_ is the peak-to-peak voltage of the AC signal). The plot shows only the reversible part of the actuation curve and does not contain the snap-down regime. The measured device is based on design S1 shown in Figure 2. (**c**) Simulated absorption spectra for different distances between the membrane and the spacer; *d*=3 μm and *d*=0 μm correspond to the undeflected and snap-down configurations, respectively. At the threshold of snap-down (*d*=2 μm), absorption changes by 80% at the initial resonant frequency of approximately 1.168 THz (marked by red circles). (**d**) Electric field amplitude on the plane of meta-atoms at the wavelength of peak absorption, normalized to incident field amplitude |*E*_0_|. The dotted line arrows indicate the direction of the surface current.

The unit cell of the tunable metamaterial absorber is schematically depicted in Figure 1a. It includes a 200-nm-thick metallic ground plane, a 2-μm-thick silicon nitride spacer, and a matrix of meta-atoms suspended above the silicon nitride layer by an adjustable distance *d*. The size of each unit cell is 180×180 μm, and each unit cell contains 9 coupled metallic meta-atoms supported by a suspended silicon nitride membrane (140 μm×140 μm×200 nm). The size of the unit cell is chosen such that it is still subwavelength at the resonance wavelength, so the diffraction will not complicate the design and characterization. The introduction of the silicon nitride membrane and spacer is crucial in reducing the footprint and thickness of the absorber due to its relatively high permittivity (*ε*_SiNx_≈7). The movable membrane is tethered to four actuation arms suspended from four posts in a square arrangement. The separation between the membrane and the spacer is *d*=3 μm at rest and can be tuned electrostatically by attracting the silicon nitride micro-beam actuators (covered by a gold top electrode layer) to the gold ground plane, which also serves as the bottom electrode (see Supplementary Figure S1 in the Supplementary Material for detailed geometries).

The MEMS actuation design utilized here is based on our prior work, and we built on our fabrication know-how accumulated during the development of a MEMS on-chip spectrometer technology for infrared wavelengths based on Fabry-Pérot wavelength discrimination^44,45,46^. Being able to harness established in-house fabrication know-how is a significant benefit to this work; however, as noted previously, although the actuation range of this MEMS design matches well with the Fabry-Pérot resonance tunability within the infrared band, the introduction of metamaterials becomes indispensable for THz applications due to the severe mismatch between the actuation range [∼1 μm of the continuous tuning range, see Figure 1b] and the THz wavelength (∼300 μm). Meta-atoms that support strongly localized resonances can significantly enhance their near-field coupling with the ground plane, leading to the ultrahigh mechanical sensitivity of the resonance (Figure 1c).

**Figure 2:**
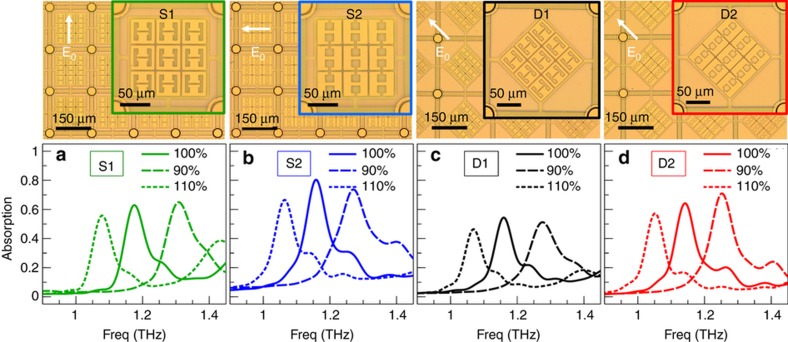
Microscope photographs and the measured THz absorption spectra of the fabricated MEMS arrays. Two designs of ESRRs (denoted as ‘1’ and ‘2’), each with two designs of membrane (‘S’ and ‘D’ denote square and diamond lattices, respectively) were fabricated. Spectra were also measured for devices with geometry scaled by 90 and 110%. The photographs correspond to the 100% footprint size design. The red arrows in the photographs show the polarization of incident electric field during absorption measurements.

To achieve polarization-dependent absorption, we selected anisotropic meta-atoms based on electric split-ring resonators (ESRRs). This type of resonator supports localized modes with circulating current and thus shows a lower radiative loss and a higher quality factor of the resonance compared to dipole-like meta-atoms such as cut-wires and I-beams^47^. We designed two different types of ESRRs (denoted as ‘1’ and ‘2’, according to the number of gaps in the meta-atoms); each design is hybridized with two different types of membrane geometries (see Figure 2, where ‘S’ and ‘D’ denote the square and diamond geometries of the membranes, respectively). Different membrane shapes were chosen to study which geometry provides a flatter membrane over the range of actuation voltages (see Supplementary Materials for further discussion). We employed CST Microwave studio (CST-Computer Simulation Technology AG, Darmstadt, Germany) to simulate the THz response of a periodic array of absorbers, with the conductivity of gold chosen as 4.56×10^7^ S m^−1^. The geometries of the devices are optimized for a plane wave under normal incidence, with the excitation polarization shown in Figure 2. As an example, the simulated THz absorption spectra for design S2 are depicted in Figure 1c. When the membrane is in the unactuated rest position (*d*=3 μm), the resonant frequency of perfect absorption (>99%) is ~1.168 THz, corresponding to a free space wavelength of ~46 times the device thickness (5.6 μm, measured from the ground plane to the meta-atom layer). The simulation shows that the electric field is strongly localized and is enhanced by >40-fold within the gaps of the meta-atoms (see Figure 1d), and ~98.5% of the energy is dissipated in the metal. The mode observed is not the resonant mode of a single meta-atom but a new hybrid mode of the group of 9 coupled meta-atoms. As the meta-atoms at different positions in the group experience different mutual coupling, the field strength is varied in the constituent meta-atoms. The simulated resonance has a linewidth (full-width half maximum) of ~47 GHz (*Q*≈24). When the membrane is actuated down by 1 μm (*d*=2 μm, corresponding to the threshold of snap-down), the resonance shifts by approximately 40 GHz, and a substantial change in absorption from nearly 100 to 20% is expected at the original resonant frequency of 1.168 THz. If the membrane is further driven to the snap-down position (*d*=0 μm), the resonance can shift by >580 GHz, and a near-perfect modulation of absorption is predicted (Figure 1c). For consistency, all of the absorption changes discussed in this paper are evaluated at the original resonant frequencies for unactuated devices.

We fabricated the MEMS tunable metamaterial absorbers on 2-inch silicon wafers using standard surface micromachining techniques that are compatible with most MEMS foundries. To facilitate characterization with a THz beam wider than the size of individual unit cells, we fabricated arrays of two sizes for each design of the absorbers, spanning nominal areas of 5×5 mm and 10×10 mm. Characterization results for both sizes of the array did not show differences, confirming that the beam width in our setup is smaller than any of the samples. The metal layers (ground planes, electrodes, and meta-atoms) were formed using electron-beam deposition of gold followed by ‘lift-off’ patterning. The structural members of the device (posts, beams, and support membranes) were formed in silicon nitride prepared via inductively coupled plasma chemical vapor deposition. These beams and support membranes were formed on top of spin-coated and patterned islands of a polyimide sacrificial layer. The polyimide islands were then removed via exposure to oxygen plasma to release the suspended structures. As the resonance is highly sensitive to the distance between meta-atoms and the ground plane, membrane bowing is detrimental to device performance. In both simulation and experiment, we noted that to achieve good device performance, the flatness of the membrane is crucial. In devices without the stress compensation layer, membrane bowing can lead to reduced peak absorption, resonance broadening and splitting due to the ultrahigh mechanical sensitivity of the resonance. By depositing a thin layer of silicon nitride with controllable in-built stress directly underneath the gold meta-atom, we were able to compensate for undesired membrane bowing in a controllable fashion. Minimal bowing of 100 nm was achieved over the 140×140 μm suspended membrane area. For more details, see Supplementary Figure S3 and the corresponding discussion in the Supplementary Material. We fabricated and measured several wafers with different thicknesses of the stress compensation layer. For consistency, all measured results shown in this paper are from the same wafer where the samples have the same thickness of the compensation layer.

## Results and discussion

To quantify the absorption properties of the samples, we measured the reflection spectra with a commercial THz time-domain spectrometer (Terapulse4000, Teraview Ltd, Cambridge, UK). The incident angle of the THz beam in the spectrometer is 30°, and the polarization is dominated by the TE component. The samples were placed in a chamber purged with nitrogen, and their orientations were adjusted to match the TE component of the incident polarization. We verified the results obtained from this system by performing measurements in another commercial THz time-domain spectrometer. Figure 2 shows the measured absorption spectra for the four designs. For the same lattice type, design 2 provides stronger absorption, and the absorption in sample S2 reaches 80%. For the same meta-atom design, the diamond lattice leads to a small-resonance redshift and a lower absorption due to the reduced filling factor of the meta-atoms. We also studied the size tunability by fabricating and measuring arrays with sizes 90 and 110% of the original unit cell designs (with no change in thickness); their corresponding resonances shifted by approximately ±10%, respectively, without a significant change in absorption (Figure 2).

Compared to the simulated absorption spectra obtained for the post-fabrication measured device geometries (Supplementary Figure S1), the experimentally measured spectra show overall good agreement (Supplementary Figure S4). Compared to the simulation results, the experimentally observed resonant frequencies are shifted, the linewidths are broadened (*Q*≈13), and the absorption is reduced. Some of these differences can be attributed to fabrication errors, nonuniformity, slight warping of the membranes, and uncertainty in the material parameters. However, a significant factor contributing to the observed discrepancies between the measured and simulated spectra originates from the experimental excitation conditions: the oblique incidence and the mixture of TE and TM polarizations lead to a further reduction in the peak absorption. The retardation introduced by oblique incidence and the TM component in the focused THz beam is also responsible for the excitation of the antisymmetric mode, corresponding to the additional small resonance peak measured at ~1.25 THz (see Supplementary Figure S5 for a more detailed discussion).

The key feature of our devices is their electromechanical tunability. To control the gap between the suspended plane of the meta-atoms and the spacer layer, we exploited the electrostatic attraction between the top actuators and the gold ground plane by employing a periodic square wave voltage (alternating between negative and positive) with a carrier frequency *f*_C_=50 kHz. The response of the devices to electrostatic actuation was monitored by measuring THz reflection spectra under different peak-to-peak voltages *V*_pp_. It is important to note that actuation of the suspended membrane is generally limited to one-third of the suspension gap. At this point of actuation, the electrostatic attraction force overpowers the mechanical restoring force, and uncontrollable snap-down prevents further downward actuation^48^. During the experiments, we increased the voltage with fine steps down to 5 V, particularly when the devices were approaching the snap-down position. Due to the slight membrane bowing and the different designs of metallic meta-atoms, membranes lattice and anchor-separation distance, the actual position of snap-down and the threshold voltage could vary for different device designs. The main reason for using a high-frequency carrier signal is to avoid the mobile charge accumulation typical for plasma-deposited dielectrics, which could result in a change in the inter-electrode electric field distribution and cause a drift in the MEMS actuator position. The carrier frequency chosen is much higher than the cutoff frequency of the mechanical response (as discussed below), and the MEMS move in response to the slow modulation with frequency *f*_M_ applied on top of the carrier (for static actuation, *f*_M_=0 Hz).

**Figure 3:**
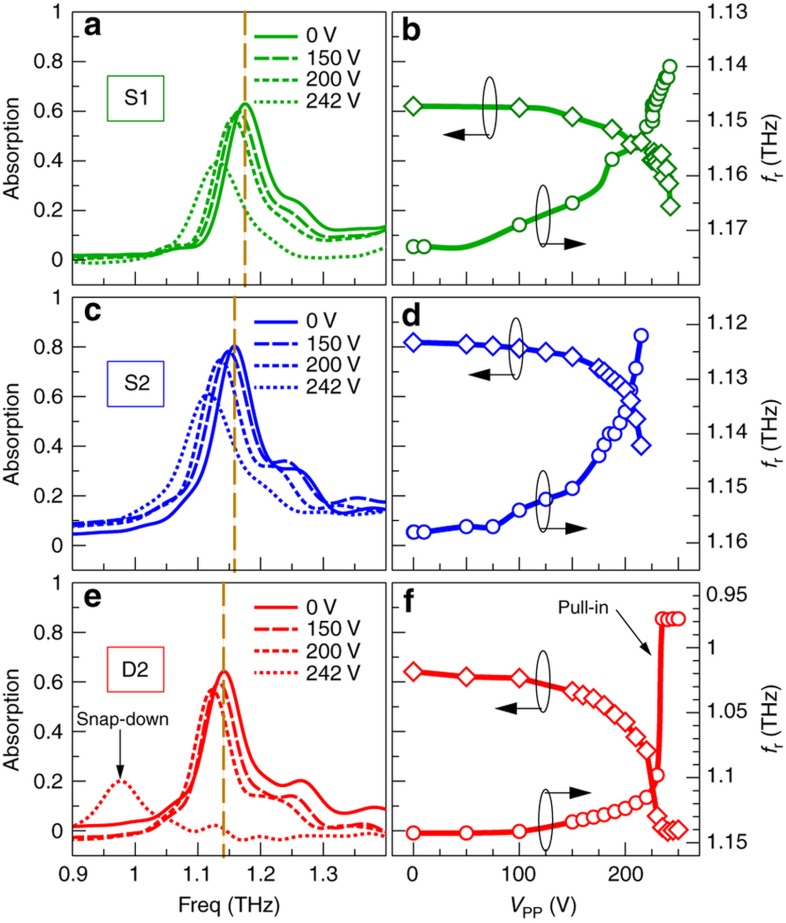
(**a**, **c**, and **e**) Measured absorption spectra for different applied peak-to-peak voltages *V*_pp_. For clarity, we only show four spectra for each sample. Devices S1 and S2 were driven up to the threshold of snap-down, whereas device D2 was driven to the snap-down state [see spectrum for 250 V in (e)]. The corresponding absorption measured at the original resonant frequencies for unactuated devices [indicated by the dashed lines in (**a**, **c**, and **e**)] and the change of resonant frequencies *f*_r_ are plotted in (**b**, **d**, and **f**).

We measured the tuning properties for three samples (100% designed footprint size) under static actuation (*f*_C_=50 kHz, *f*_M_=0), as shown in Figure 3. For clarity, we only plot four spectra for each device. Because an anti-stiction layer was not employed in the presented devices, the arrays suffered from stiction after being driven to snap-down. To achieve repeatable measurements, devices S1 and S2 were only tested below the threshold voltage of snap-down, whereas device D2 was driven to snap-down to test the maximum modulation. All three devices show reasonably large resonance shifts as the membranes approach the threshold of snap-down (∼45 GHz (∼56% of the resonance linewidth) for S1 and S2, and ∼30 GHz (∼38% of the resonance linewidth) for D2). Measured at the original resonant frequency of for the unactuated device, an absolute change in absorption of ~40% is achieved for S1 and S2, and 32% for D2 (Figures 3b, d, and f); the corresponding relative change of absorption is ~65% for S1, and 51% for S2 and D2. The measured resonance shifts at the threshold of snap-down are consistent with the full wave simulation for the same actuation distance (*δd*∼1 μm) measured by an optical profiler, corresponding to a mechanical sensitivity *δλ*_0_*/δd*≈9.

To test the maximum modulation, we increased the voltage above the snap-down threshold and drove device D2 to the snap-down state (see spectrum for 250 V in Figure 3e). The resonance shifts by 165 GHz (∼206% of the resonance linewidth, 14.4% of initial resonance wavelength, *δλ*_0_*/δd*≈14.8) and a change of absolute absorption of up to 65% is achieved (Figures 3e and f). For a resonance shift of >200% of the resonance linewidth, the relative modulation of absorption at the initial resonant frequency is already nearly 100%, and the absolute absorption change could be further enhanced with an excitation condition matching the simulation, as discussed above. Such large shifts in the resonance have been observed for other arrays that were driven to snap-down (not shown here). We note that for some measurements, the background absorption can go slightly below zero, and we can attribute this value to possible humidity changes or mechanical instabilities during measurement, leading to the normalized reflectance being slightly greater than one due to imperfect system calibration. Compared to the simulation, the measured resonance shift at the snap-down state is smaller. One possible explanation for this result is incomplete snap-down; i.e., a small gap forms between the membrane and the top of the spacer due to imperfections in fabrication. Because both the electromechanical and electromagnetic responses become highly nonlinear in this regime, i.e., the displacement of meta-atoms is a strong nonlinear function of the applied voltage, the resonant wavelength is highly nonlinear with respect to the displacement; further study is required to identify the underlying reason.

**Figure 4:**
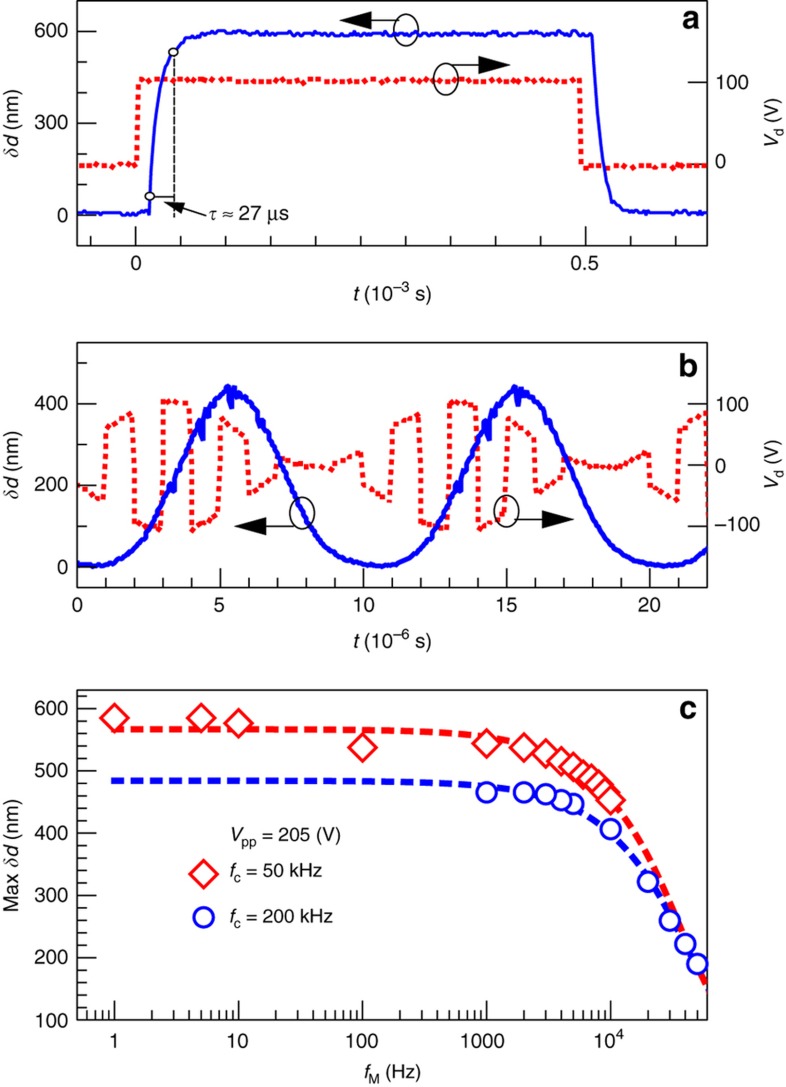
Dynamic response of mechanical displacement (red dotted line). (**a**) *f*_C_=1 kHz, *f*_M_=0 Hz; (**b**) *f*_C_=50 kHz, *f*_M_=10 kHz. The corresponding driving signal *V*_d_ (blue solid line). (**c**) Maximum displacement for two different carrier frequencies *f*_C_ as a function of modulation frequency *f*_M_. The points depict actual measurements, and the dashed curves depict a fit to the measurement data using an exponential decay model and a single time constant.

To understand the dynamic mechanical response, we monitored the displacement of the meta-atoms using an optical vibrometer. We first used a 1 kHz square wave (0 to 100 V) to drive sample S1. The time-dependent displacement shows that the device is over-damped at ambient air pressure, and the response time, measured from 10 to 90% displacement, is *τ*≈27 μs (Figure 4a). For a single pole system, the 3 dB bandwidth is related to the rise time by BW=0.35*/τ*, so the bandwidth estimated from the rise time is approximately 12.9 kHz.

Subsequently, the square wave carrier (*f*_C_=50 kHz) was modulated by a sinusoidal wave with frequency *f*_M_. Figure 4(b) shows the device response under a modulation frequency of 10 kHz, where a slight decrease in the displacement amplitude was observed compared to the case of *f*_M_=0 shown above. By comparing the modulation signal and the displacement signal in Figure 4b, it is evident that the displacement amplitude of the suspended membrane follows the modulation signal adequately and repeatedly. To check the effect of carrier frequency *f*_C_ and modulation frequency *f*_M_ on the displacement amplitude, we drove the device with a fixed actuation voltage (*V*_pp_=205 V). The maximum displacement for two different carrier frequencies as a function of modulation frequency is summarized in Figure 4c. The displacement decreases as the carrier frequency changes from 50 to 200 kHz. This drop in displacement amplitude with carrier frequency is likely due to inadvertent low-pass filtering in the drive circuitry. For the 50 kHz carrier frequency, the mechanical displacement decreases by approximately 23% as *f*_M_ increases from 1 Hz to 10 kHz, after which the response starts to cut off. We note that the data measured for both carrier frequencies of 50 and 200 kHz show a 3 dB high-frequency cutoff at ~16.5 kHz. Given experimental errors, this result is in reasonably good agreement with the bandwidth (12.9 kHz) based on the 27 μs time constant from Figure 4a.

The voltages used for MEMS actuation in this work extend to >200 V; however, we want to emphasize that these are peak-to-peak voltage values for the adopted AC actuation and that the corresponding RMS voltage values that need to be used for comparison with direct current actuation are significantly less striking. Nevertheless, for system integration, it is desirable to actuate with lower voltages, as we have achieved for similar actuation geometries (for example, actuation under 24 V (Ref. 49) or 8 V (Ref. 50)). Compared to our prior work, the relatively high actuation voltages required here are mostly due to the actuation electrodes being separated much further than in the past. The unactuated electrode separation gap in this work is >5 μm compared with >1.5 μm reported in Ref. 50, and for a given voltage, the electrostatic attraction force between the electrodes is approximately inversely proportional to the square of mutual separation. To decrease the actuation voltage requirements, future design iterations could include more flexible actuators, greater electrode area overlap, and/or a design where the gap between the electrodes is de-coupled and not necessarily equal to the separation between the suspended meta-atom membrane and the gold ground plane.

## Conclusion

In summary, we presented an experimental study of ultrathin tunable THz absorbers based on MEMS-driven metamaterials. Utilizing the high mechanical sensitivity of thin metamaterial absorbers, we proposed a paradigm to combine meta-atoms and suspended flat membranes to simultaneously maximize the near-field coupling and avoid resonance broadening. We employed MEMS technology and successfully fabricated THz absorbers based on the integration of meta-atoms and MEMS, demonstrating an extremely wide tuning range of resonant absorption. The devices presented in this paper are among the best-performing tunable THz absorbers achieved to date^32,51,52^, particularly with respect to device thickness and tunability. As the first prototype device, further optimization, including enhancement of maximum absorption, reduction of operation voltage, and introduction of an anti-stiction layer^53^, is required before practical applications. The current design facilitates integration with addressable circuits to realize individual pixel control in the array, which is particularly attractive for sophisticated applications relying on the full spatial control of THz waves^11, 54,55,56^.
