# Supplementary material for: Ultrathin tunable terahertz absorber based on MEMS-driven metamaterial
Source: Microsyst Nanoeng. 2017 Aug 28;3:17033. doi: 10.1038/micronano.2017.33 (PMC6445006; doi:10.1038/micronano.2017.33)
Supplement: Supplementary Information [file micronano201733-s1.pdf]

Supplementary file

Ultra-thin tunable terahertz absorber based on MEMS-driven metamaterial

Mingkai Liu[1], Mohamad Susli[2], Dilusha Silva[2], Gino Putrino[2], Hemendra Kala[2], Shuting Fan[3], Michael Cole[1], Lorenzo Faraone[2], Vincent P. Wallace[3], Willie J. Padilla[4], David A. Powell[1], Ilya V. Shadrivov[1] and Mariusz Martyniuk[2]

Microsystems & Nanoengineering (2017) **3,** 17033; doi:10.1038/micronano.2017.33; Published online: 28 August 2017

DESIGNED AND POST-FABRICATION MEASURED GEOMETRIES OF META- MATERIAL TUNABLE ABSORBERS

We designed metamaterial tunable absorbers based on the integration of electric split-ring resonators (ESRRs) and micro-electro-mechanical-systems (MEMS) technologies. Here we use two different types of ESRRs (denoted as "1" and "2", respectively, according to the number of gaps in the meta-atom), each with two types of membrane designs (denoted as "S" and "D" for the square and diamond lattices, respectively). After fabrication, we measured the geometries of the samples under an optical microscope. The designed and the measured geometries are summarized in the caption of Supplementary Figure S1.

FABRICATION OF MEMS-DRIVEN METAMATERIAL TUNABLE ABSORBERS

The samples were fabricated on 2-inch silicon wafers using standard surface microma- chining techniques compatible with most MEMS foundries. For each design, multiple arrays of MEMS metamaterial absorbers were fabricated [25×25 (50×50) units for square lattice and 20×20 (40×40) units for diamond lattice arrays for a nominal array size of 5×5 mm (10×10 mm)]. Supplementary Figure S2a is the photograph of one of the fabricated wafers. For array electrostatic actuation, the electrodes are bonded to a circuit board with gold wires, and the samples were driven with a voltage amplifier, connected to a signal generator.

The top moving structure of the MEMS devices consisted of a silicon nitride membrane with meta-atoms deposited on top. However, the initially fabricated devices depicted a significant bowing of over 1 μm, as shown in Supplementary Figure S3a, due to the in-built stress mismatch between the gold meta-atoms and the silicon nitride membrane. This bowing would degrade the device performance, leading to reduced peak absorption, broadened linewidth and excitation of additional resonances (see the comparison in Supplementary Figure S3b). In order to flatten these devices, some degree of stress compensation was necessary. A compressive stress compensation layer was introduced in between the silicon nitride membrane and the gold meta-atoms to achieve the desired flattening. In order not to affect the THz properties of the silicon nitride membrane, the compensation layer was also composed of silicon nitride. As such, we used an alternative deposition recipe for silicon nitride, which was known to produce compressive films (film stress approximately 1 GPa). The required thickness of the compressive layer was ascertained by trial-and-error, where the deposition time was varied between the trials. An adequately low level of bowing around 100 nm was achieved by this method, which was sufficiently flat for the needs of the application (Supplementary Figure S3c–e).

The diamond shape design was used along with the square shape for the benefits of mechanical stability under stress imbalance. We were expecting the stress imbalance between the meta-atoms and the supporting membrane layer to warp the square membrane to some extent. In comparison to the square membrane, the same stress imbalance would warp a diamond membrane to a smaller degree, if one is happy to sacrifice on the fill factor. We measured the membrane flatness with optical surface profiler and did observe the diamond membranes to warp to a smaller extent (~1.4 μm) than the square membranes (~5.5 μm) in the first fabrication run without the stress compensation layer, and this warping was eliminated with the introduction of the compensation layer in the second run.

COMPARISON OF MEASUREMENT AND SIMULATION

The characterization of Thz reflection spectra was performed with a commercial THz time-domain spectrometer (Terapulse4000, Teraview Ltd, Cambridge, UK). During the THz characterization, we first scanned the wafer, and the location of the absorber arrays can be clearly identified via the observed reduction in reflection (see Supplementary Figure S2b). Subsequently, we performed high-resolution measurements at the location of the absorber arrays by increasing the integration time. The angle of incidence of the focused THz beam is 30°. The polarization of the THz beam is dominated by TE polarization (~88% at around 1 THz, based on the data provided by Teraview). The wafer was rotated in order to match the dominant TE polarization of the THz source and the orientation of meta-atoms.

Due to the resolution limit in micro-fabrication, the widths of meta-atom features are slightly larger than the designed ones (see measured values in the caption of Supplementary Figure S1), and the effectively reduced meta-atom gap size leads to a red-shift of resonance. Using the measured geometries instead of the designed ones to re-simulate the spectra under normal incidence excitation, we achieve a reasonably good agreement between the measured and simulated spectra (see Supplementary Figure S4). The sharp features in the simulated spectra for D1 and D2 around 1.175 THz are due to the interference of the first order diffraction mode (the Wood's anomaly) from the diamond lattice, which has a larger period compared to the square lattice.

[1]Nonlinear Physics Centre, Research School of Physics and Engineering, Australian National University, Canberra, ACT 2601, Australia; [2]School of Electrical, Electronic and Computer Engineering, the University of Western Australia, Crawley, WA 6009, Australia; [3]School of Physics, University of Western Australia, Crawley, WA 6009, Australia and [4]Department of Electrical and Computer Engineering, Duke University, Durham, North Carolina 27708, USA
Correspondence: Mingkai Liu (mingkai.liu@anu.edu.au)

However, such sharp features were not resolved in the experiment due to several reasons: (1) the finite sample size which limits the formation of high Q factor collective mode with long spatial coherence length; (2) the limited spectral resolution in the measurement; (3) the incident wave is a focused beam instead of a plane wave, and the wavelength of Wood's anomaly is highly sensitive to the angle of incidence, thus the sharp feature was smoothened after angle-averaging of the collected signal.

However, we notice that the fabrication error is not sufficient to fully explain the difference between the simulated and measured spectra, particularly the lower absorption and the additional small resonance around 1.25 THz. To have a better understanding, we simulate the absorption spectra of device S2 (100% footprint size) under different angles of incidence and polarizations, as denoted in Supplementary Figure S5a. We first keep the orientation angle $\varphi = 0$ and simulate the effect of the angle of incidence θ using TE polarization. As depicted in Supplementary Figure S5b, the original resonant frequency around 1.16 THz shows little changes as the angle of incidence increases; however, a new resonance exists around 1.127 THz due to the excitation of a hybrid mode of different symmetry. The peak absorption decreases from near 100% at normal incidence to around 90% at $\theta = 30°$. This is clearly one of the reasons of the reduced peak absorption in measurement. Next, we change the orientation angle $\varphi = 90°$ and calculate spectra under different angles of incidence for TM polarization. Once $\theta \neq 0$, a new resonance around 1.3 THz is excited and becomes quite noticeable as θ increases. Since in the experiment, we used a focused THz beam, and the polarization is not purely TE, the TM component of the focused beam with wave vector $\varphi \neq 0$ partially contributes to the measured spectra. Considering the red-shift of the measured resonances compared to the simulated ones, this resonance around 1.3 THz in simulation matches well with the small resonance around 1.25 THz measured in the experiment.

Considering the simulation results we can identify the symmetry of the modes. Supplementary Figure S5c plots the surface current component J_x of the two modes excited under oblique incidence ($\theta = 30°$, $\varphi = 90°$), as denoted by the circle and diamond in Supplementary Figure S5b. For clarity, we show the current in two adjacent unit cells. For the dominant mode around 1.16 THz, the current of the meta-atoms in each unit cell oscillates almost in phase, while for the mode around 1.3 THz there is an antisymmetric pattern; by symmetry, it can only be excited at oblique incidence via retardation effect.

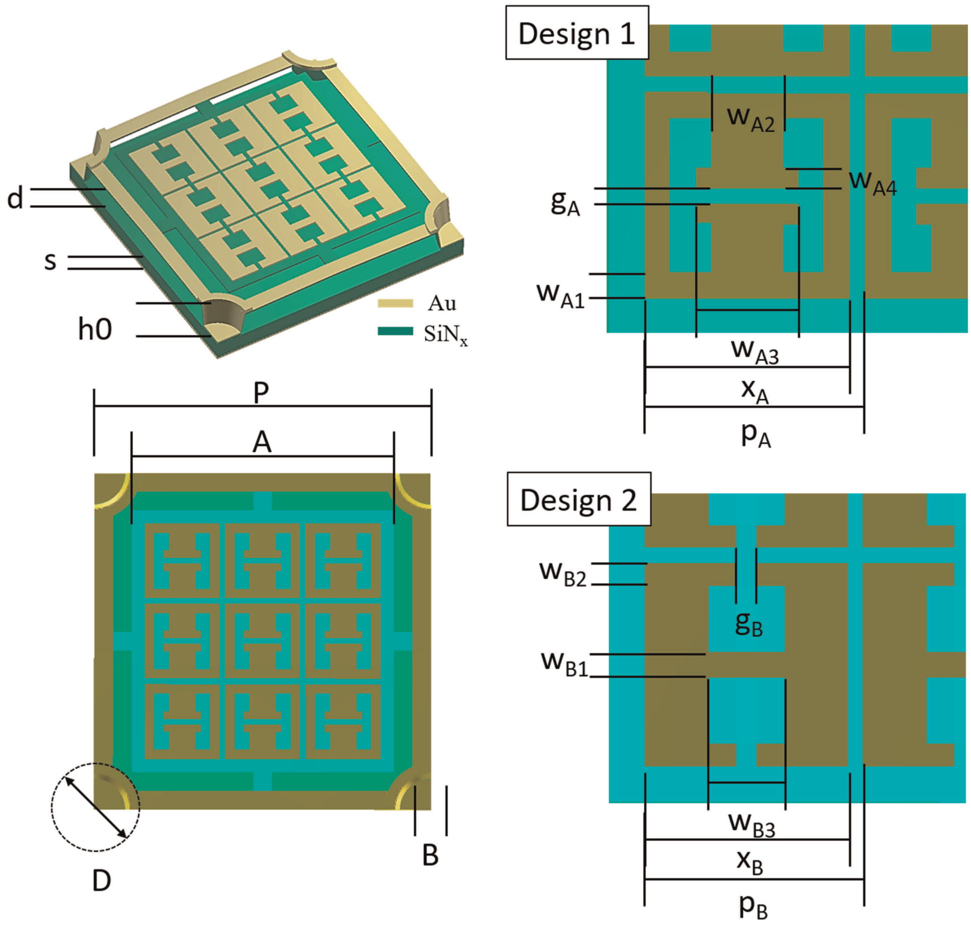

Figure S1 Details of the designed (post-fabrication measured) geometries of the metamaterial absorbers. All sizes are given in μm. For 100% footprint size design: $h_0 = 3$, $s = 2$, $d = 3$ (~2.9) at rest; $P = 180$, $A = 140$, $D = 50$, $B = 20$; $g_A = 3$ (~2.2), $w_{A1} = 5$ (~6.1), $w_{A2} = 14$ (~15.1), $w_{A3} = 20$ (~22.1), $w_{A4} = 4$ (~5.4), $x_A = 40$ (~41.3), $p_A = 43$ (~43.2); $g_B = 3$ (~3.1), $w_{B1} = 5$ (~6.0), $w_{B2} = 4.5$ (~5.3), $w_{B3} = 15$ (~14.2), $x_B = 40$ (~41.1), $p_B = 43$ (~43.3); the thicknesses of silicon nitride membrane and gold meta-atoms are both 0.2.

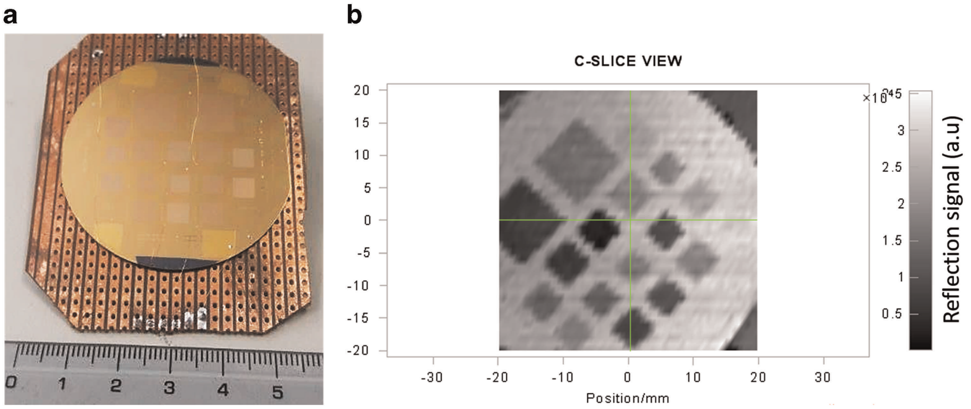

Figure S2 (**a**) Photograph of the fabricated sample; (**b**) Wafer scanned with a THz spectrometer (Teraview), dark areas show the position of metamaterial absorbers.

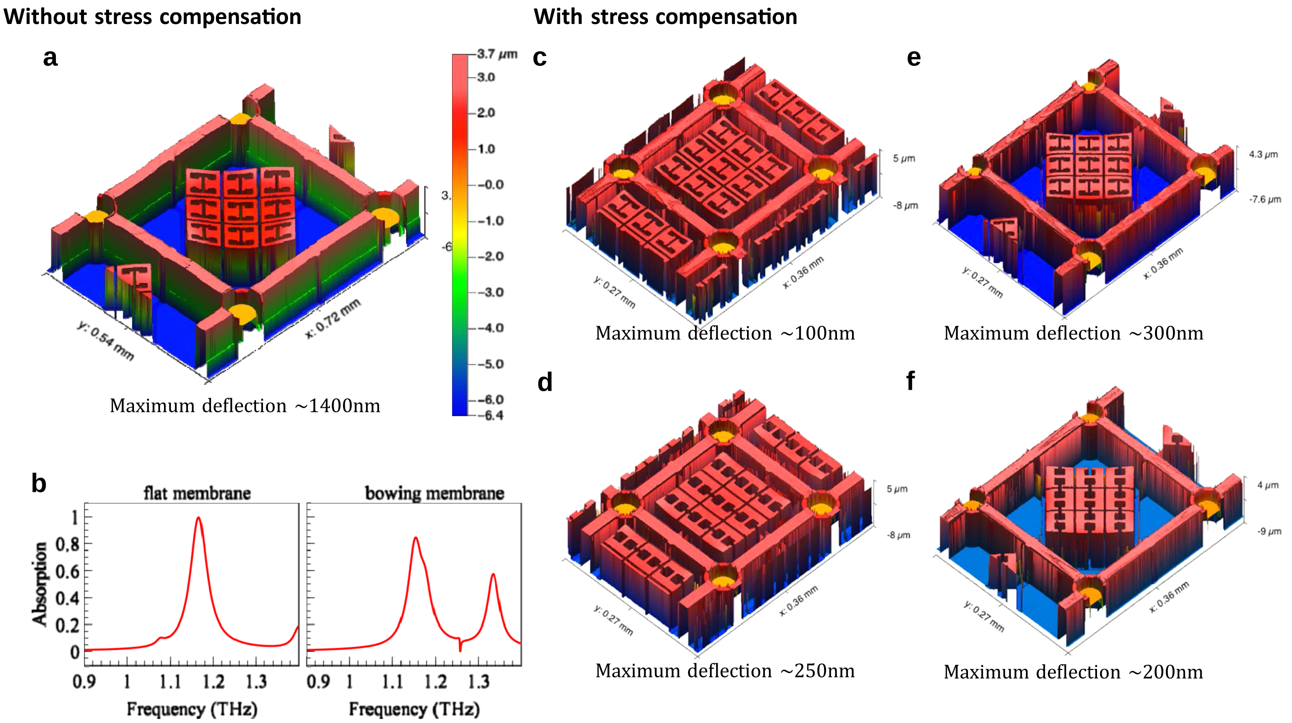

Figure S3 Surface profiles of the metamaterial absorbers measured with the optical profiler. Comparison of (**a**) and (**c**–**f**) shows that the bowing of membrane is significantly reduced after using a stress compensation layer. Note that (**a**) shows the bowing in a diamond lattice device which was adopted to reduce membrane bowing in comparison to square lattice devices, which without stress compensation bowed to a greater extent. (**b**) Simulated absorption spectra of design S2 (100% footprint size) at rest position under normal incidence. The left plot is for a perfectly flat membrane, while the right one is for a bowing membrane. The bowing surface profile is measured by an optical surface profiler, with the maximum bowing of 5.5 μm measured from the center to the edge of the membrane.

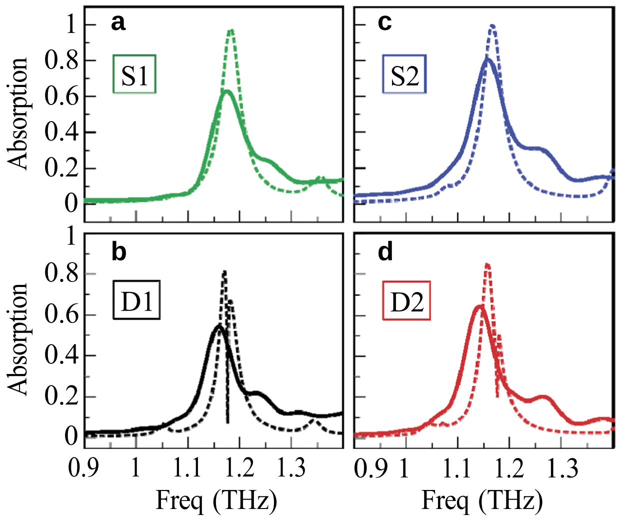

Figure S4 Comparison of the simulated (dashed curves) and measured (solid curves) spectra of the 100% design footprint size absorbers for suspended membranes in the unactuated rest position.

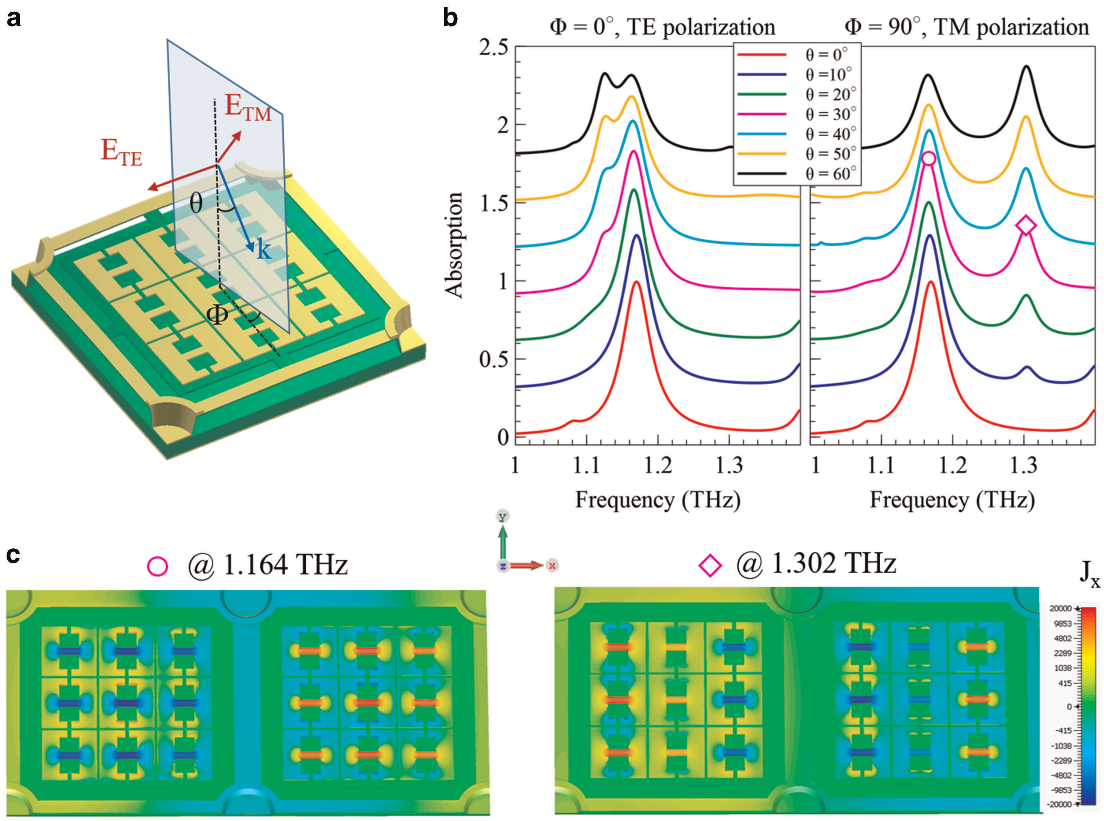

Figure S5 (**a**) Schematic of metamaterial absorber under oblique incidence. (**b**)Simulation absorption spectra of device S2 (100% footprint size) under different angles of incidence and polarizations. (**c**) The snap-shots of the surface current component J_x of the two different modes excited under oblique incidence ($\theta = 30°$, $\varphi = 90°$), denoted by the circle and diamond in the spectrum of TM polarization excitation. For clarity, curves are offset by 0.3 in sequence.
